# Supplementary material for: Multi-trait meta-analyses reveal 25 quantitative trait loci for economically important traits in Brown Swiss cattle
Source: BMC Genomics. 2019 Sep 3;20:695. doi: 10.1186/s12864-019-6066-6 (PMC6724290; doi:10.1186/s12864-019-6066-6)
Supplement: Supplementary file 1 — Table S1. Number of Brown Swiss bulls for 56 economically important traits and their classification. (DOCX 19 kb) [file 12864_2019_6066_MOESM1_ESM.docx]

Additional file 1: Table S1 Number of Brown Swiss bulls for 56 economically important traits and their classification.

| Trait category | Trait name | N of Animal | |
| --- | --- | --- | --- |
|  |  | Swiss | Austrian-German |
| Milk production (n=10) | Milk yield | 1874 | 2497 |
|  | Fat yield | 1874 | 2497 |
|  | Protein yield | 1874 | 2497 |
|  | Fat percentage | 1874 | 2497 |
|  | Protein percentage | 1874 | 2497 |
| Body Size (n=24) | Stature | 1875 | 2389 |
|  | Chest width | 1873 | 2279 |
|  | Body Depth | 1874 | 2389 |
|  | Angularity | 1871 | 2381 |
|  | Rump length | 1874 | 2181 |
|  | Rump width | 1874 | 2387 |
|  | Rump angle | 1874 | 2389 |
|  | Thurl position | 1872 | 1443 |
|  | Backline | 1864 | 2387 |
|  | Overall frame | 1867 | 2182 |
|  | Overall rump | 1850 | 1443 |
|  | Final conformation Score | 1861 | 1414 |
| Leg conformation (n=10) | Hock angularity | 1866 | 2389 |
|  | Hock development | 1862 | 2387 |
|  | Foot angle | 1850 | 2389 |
|  | Hoof height | 1837 | 2386 |
|  | Overall leg conformation | 1854 | 2382 |
| Mammary gland morphology (n=26) | Fore udder length | 1865 | 2387 |
|  | Rear udder width | 1863 | 2389 |
|  | Rear udder height | 1865 | 2389 |
|  | Suspension ligament | 1861 | 2389 |
|  | Udder depth | 1872 | 2389 |
|  | Fore udder attachment | 1866 | 1384 |
|  | Udder balance | 1865 | 1341 |
|  | Teat length | 1872 | 2389 |
|  | Teat thickness | 1872 | 1369 |
|  | Teat placement (front) | 1872 | 2156 |
|  | Teat placement (rear) | 1871 | NA |
|  | Teat direction (rear) | 1870 | 2387 |
|  | Udder cleanness | NA | 2381 |
|  | Overall udder score | 1861 | 2384 |

**Table S1 Number of Brown Swiss bulls for 56 economically important traits and their classification (continued).**

| Trait type | Trait name | N of Animal | |
| --- | --- | --- | --- |
|  |  | Swiss | Austrian-German |
| Fertility (n=5) | Non-return rate (heifer) | 1811 | NA |
|  | Non-return rate (cow) | 1836 | NA |
|  | Interval from first to last insemination (heifer) | 1801 | NA |
|  | Interval from first to last insemination (cow) | 1820 | NA |
|  | Days to first service | 1809 | NA |
| Calving (n=9) | Direct / maternal calving ease | 1632 / 1532 | 2530/2526 |
|  | Direct / maternal birth weight | 1639 / 1747 | NA |
|  | Direct / maternal gestation length | 1768 / 1776 | NA |
|  | Direct live birth | 1068 | NA |
| Growth and carcass quality (n=8) | Daily net gain | 1470 | 2529 |
|  | Carcass percentage | NA | 2460 |
|  | Carcass grading | 1450 (CHATX) | 2527 (EUROP) |
|  | Daily net gain (calf) | 1527 | NA |
|  | Carcass grading (calf) | 1480 (CHATX) | NA |
|  | Milking speed (n=2) | 1846 | 2517 |
|  | Longevity (n=2) | 1805 | 2529 |
|  | Persistency (n=2) | 1868 | 2530 |
|  | Somatic cell score (n=2) | 1861 | 2517 |

*n* number of traits included in trait-category meta-analysis
