# Supplementary figures and images for: Multi-trait meta-analyses reveal 25 quantitative trait loci for economically important traits in Brown Swiss cattle
Source: BMC Genomics. 2019 Sep 3;20:695. doi: 10.1186/s12864-019-6066-6 (PMC6724290; doi:10.1186/s12864-019-6066-6)

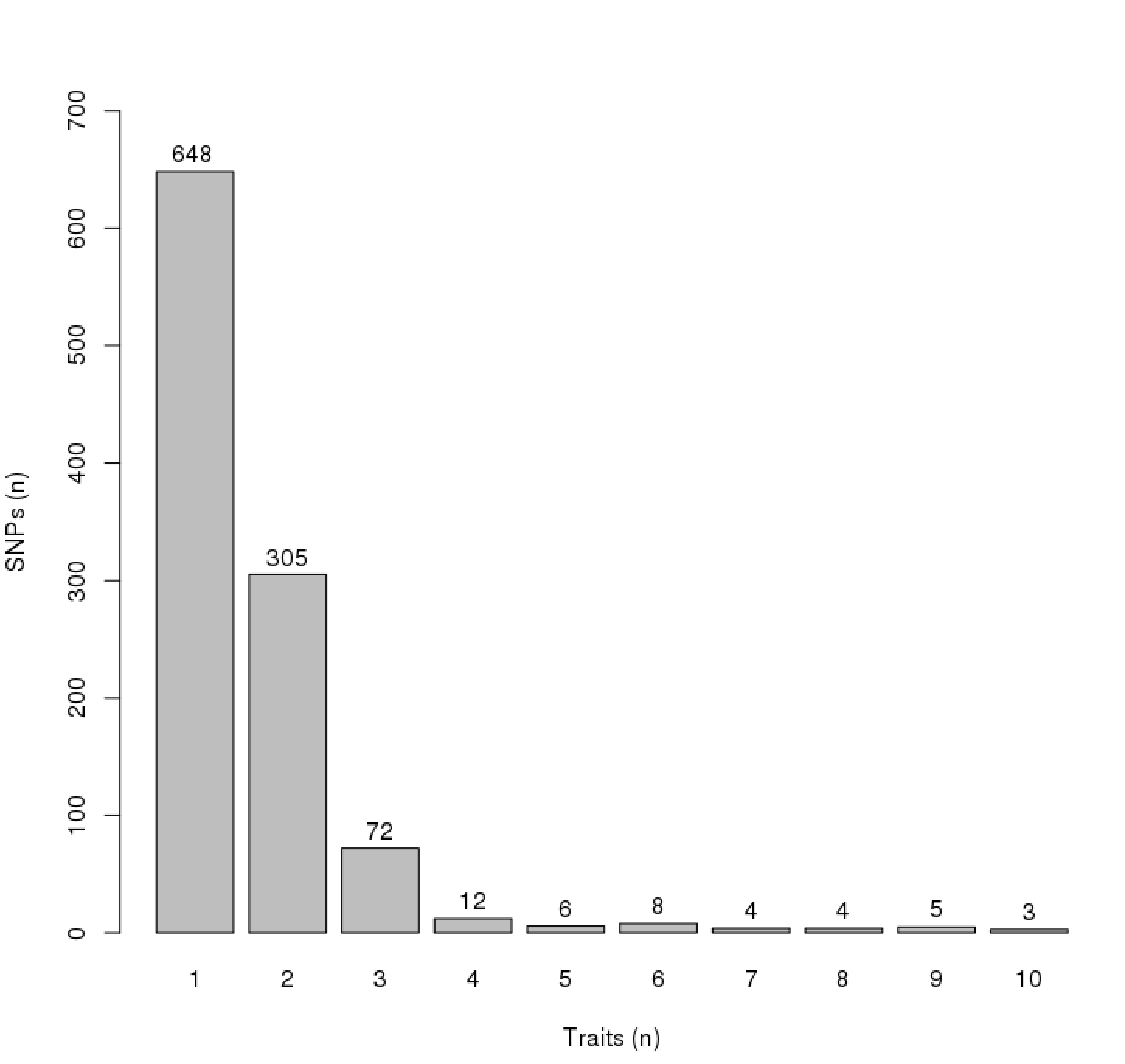

Supplement: Supplementary file 2 — Figure S1. Distribution of significant SNPs by number of traits affected for each of 56 economically important traits. (PNG 62 kb) [file 12864_2019_6066_MOESM2_ESM.png]

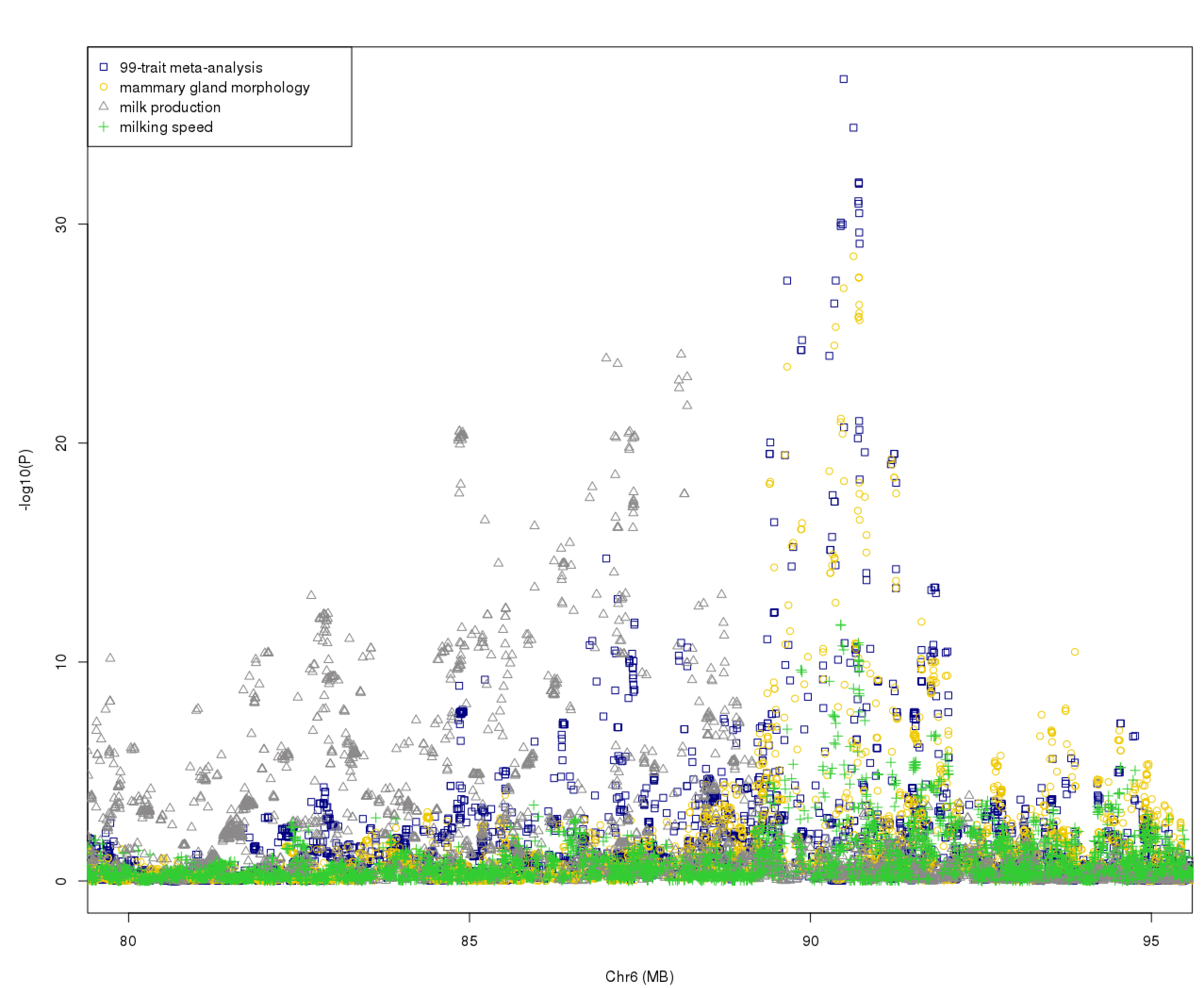

Supplement: Supplementary file 3 — Figure S2. QTL signals detected from milk production, mammary gland morphology and 99-trait meta-analysis on chromosome 6. (PNG 581 kb) [file 12864_2019_6066_MOESM3_ESM.png]

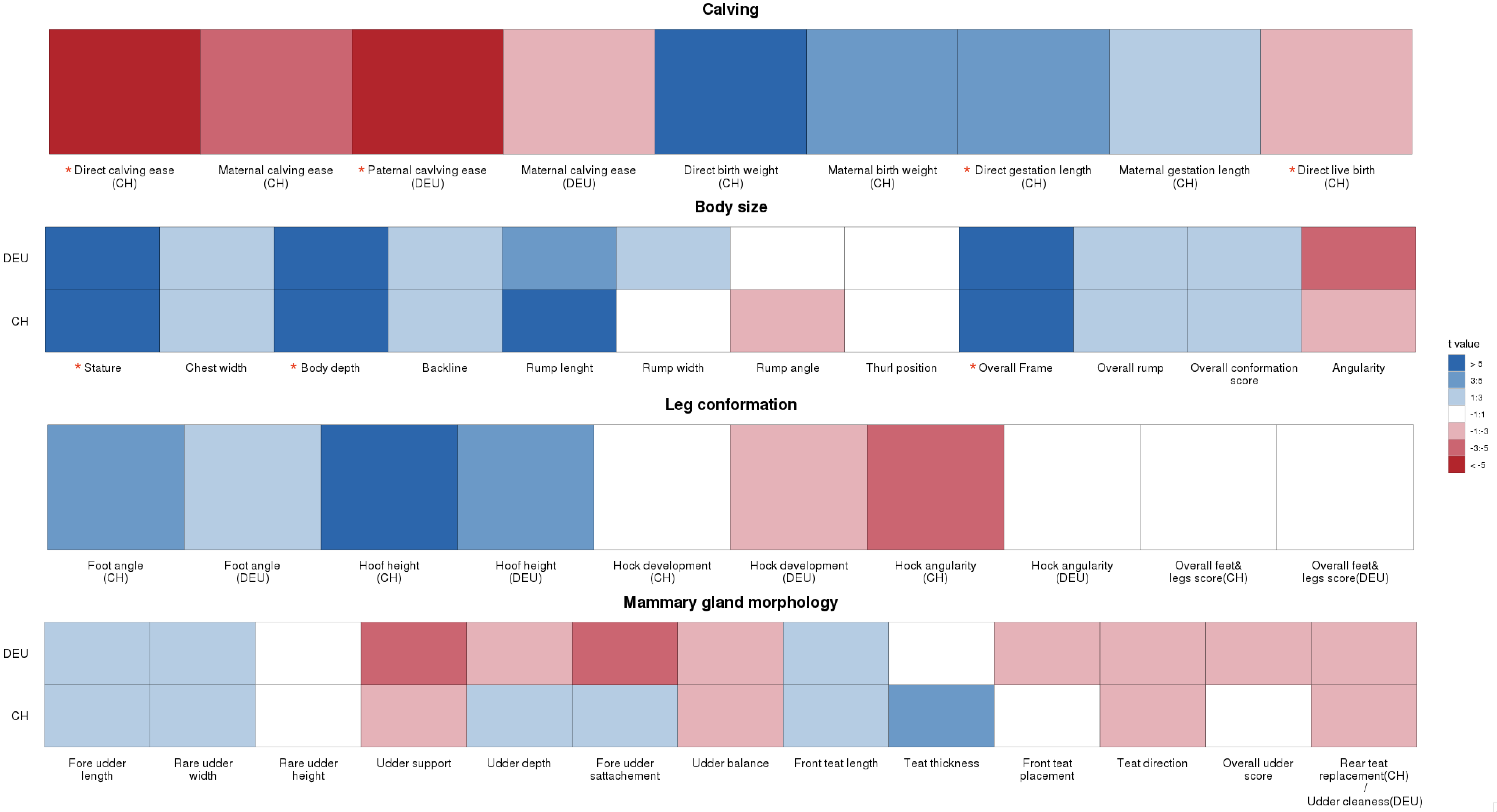

Supplement: Supplementary file 5 — Figure S3. Effects of the lead SNP of the QTL on chromosome 25 across traits. CH: Swiss population; DEU: Austrian-German population. Effects are t-values estimated from single-trait associations. (PNG 241 kb) [file 12864_2019_6066_MOESM5_ESM.png]

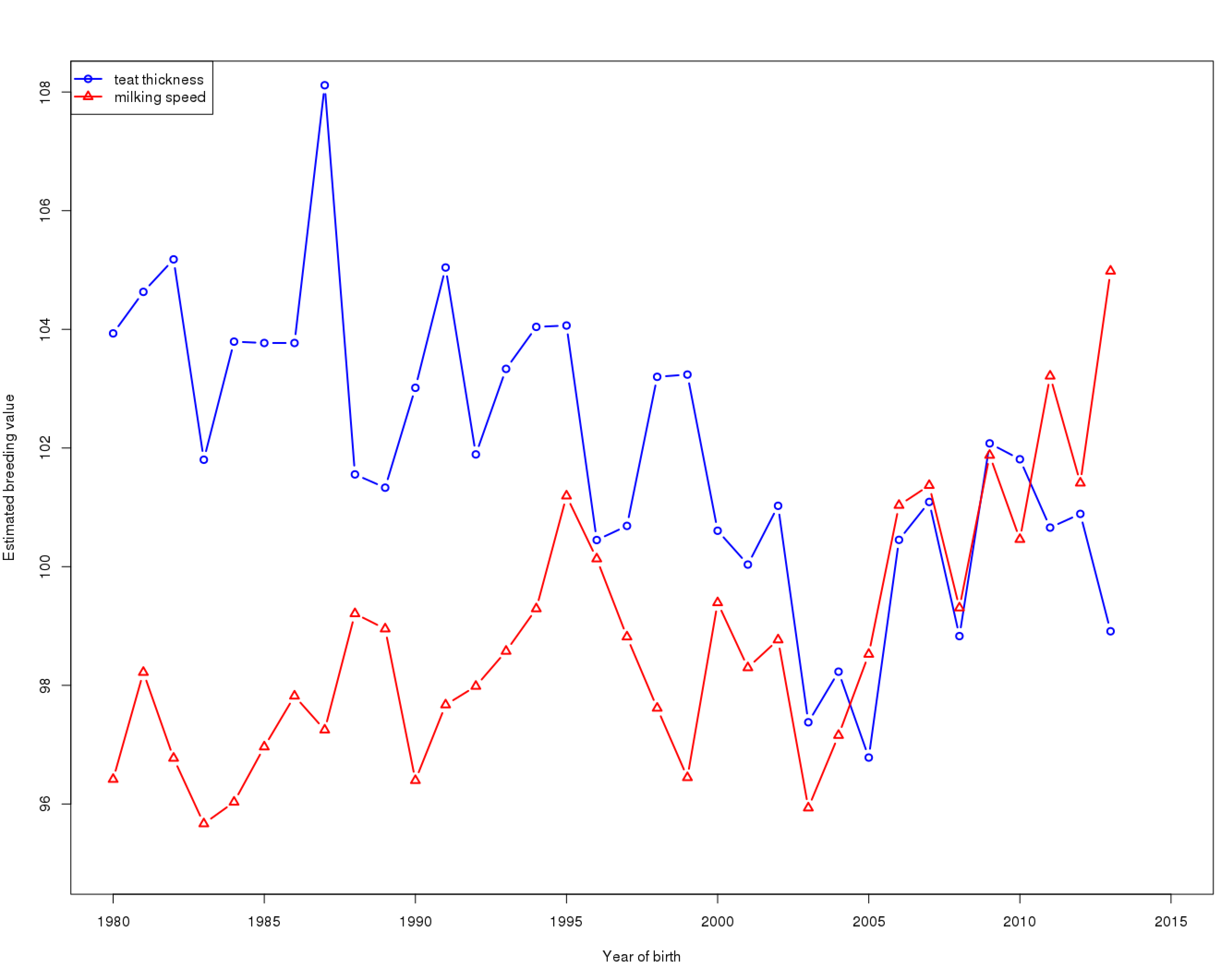

Supplement: Supplementary file 6 — Figure S4. Genetic trends for teat thickness and milking speed from year 1980 to 2015. (PNG 232 kb) [file 12864_2019_6066_MOESM6_ESM.png]

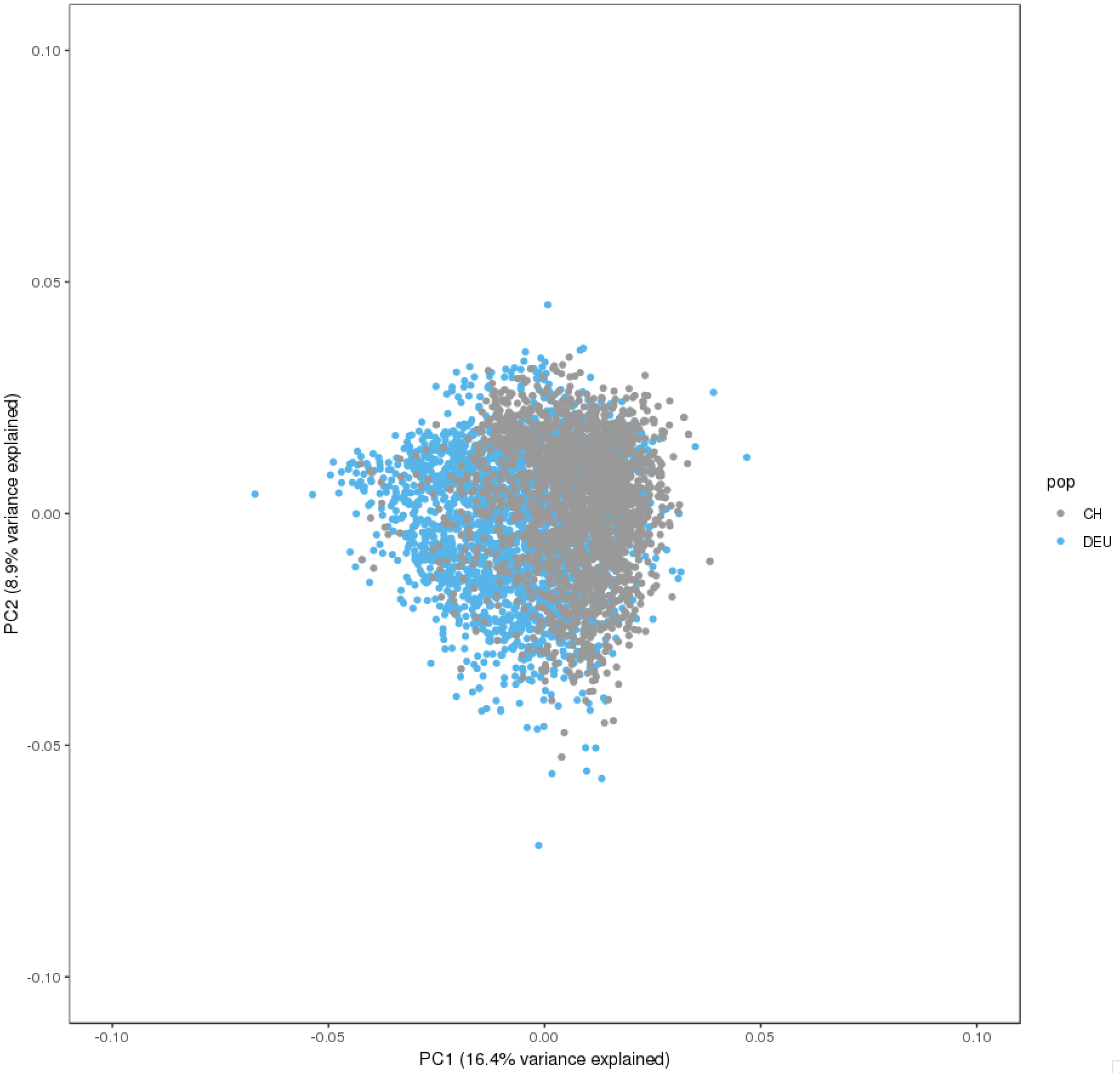

Supplement: Supplementary file 7 — Figure S5. Principle component analysis to evaluate the extent of population structure using genomic relationship matrix comprising 4758 bulls. CH: Swiss population; DEU: Austrian-German population. (PNG 187 kb) [file 12864_2019_6066_MOESM7_ESM.png]
